# Early Impact and Performance Characteristics of an Established Anal Dysplasia Screening Program: Program Evaluation Considerations

**DOI:** 10.2174/1874613600701010011

**Published:** 2007-11-29

**Authors:** Christopher Mathews, Joseph Caperna, Edward R. Cachay, Bard Cosman

**Affiliations:** 1 Department of Medicine , University of California, San Diego, CA, USA; 2 Department of Surgery , University of California, San Diego, CA, USA

**Keywords:** Anal dysplasia, screening, HIV.

## Abstract

**Background:**

Screening for invasive anal cancer and its precursors is being increasingly advocated as a response to increasing incidence among HIV-infected persons. We implemented a comprehensive screening program in 2001 and report our early experience to inform monitoring and evaluation of such programs. Our research aims were: (1) to estimate incidence of and mortality from invasive anal cancer (IAC) before (1995-2000) and after (2001-2005) screening program implementation and (2) to examine potential screening program quality indicators.

**Methods:**

The study cohort included all patients under care for HIV infection at UCSD Owen Clinic between 1995-2005. Person-time incidence rates (IR) and case survival of IAC were estimated for the pre-screening (1995-2000) and post-screening (2001-2005) periods. High resolution anoscopy (HRA) operator accuracy was estimated by kappa agreement between cyto-histologic comparisons. Program quality indicators included: (1) screening coverage; (2) percent technically unsatisfactory cytology smears; (3) time between 1st abnormal cytology and 1st HRA; and (4) time between last clinic visit and last cytology.

**Results:**

28 cases of IAC and 13,411 person-years were observed between 1995-2005. IRs (95% CI) pre-screening and post-screening were 199 and 216 per 100,000 person-years, respectively. There was no routine treatment of high grade squamous intraepithelial lesions (HSIL) during the study period. The percent of patients with IAC requiring chemoradiation decreased from 90.9% to 70.6% (p=0.36). There was a significant improvement in cyto-histologic agreement at HRA with increasing operator experience (r=0.92, p=0.025). Screening coverage was 73% of the target population. Among 14 providers, the percent unsatisfactory cytology smears averaged 27% but varied from 0 – 62%. The median time from 1st abnormal cytology to 1st HRA was 258 days. The median interval between the last cytology and the last clinic visit was 207 days.

**Conclusion:**

(1) The overall IR of IAC did not decline in the screening era and was higher than previous estimates for HIV cohorts; (2) stage shift to IAC of more favorable prognosis is a reasonable screening goal; (3) HRA accuracy varied by provider experience; (4) because of delay in access to HRA, digital rectal exam should be combined with cytology screening to detect palpable disease.

## BACKGROUND

Screening for invasive anal squamous cell carcinoma and its precursors has been increasingly advocated in high risk populations, especially HIV infected men having sex with men. Although the most recent U.S. Public Health Service guidelines for prevention of opportunistic infections[1] do not recommend routine screening, the New York State Department of Health AIDS institute recently recommended baseline and annual anal cytology examinations with referral for high resolution anoscopy and/or biopsy for cytology abnormalities for: men who have sex with men, any patient with a history of anogenital condylomas, and women with abnormal cervical/vulvar histology [2]. The present state of knowledge to justify such a screening program was recently reviewed [3, 4]. In 2001, we implemented a comprehensive screening program for anal cancer and its precursor lesions in the UCSD Owen Clinic, an academic multidisciplinary adult HIV clinic in San Diego and have previously presented our observations regarding the prevalence of detected abnormalities, their association with degree of immunosuppression, and the reproducibility of screening component measurements [5-7]. In the current work, we present preliminary findings regarding implementation of our screening program and discuss challenges to scientific evaluation of such a screening program using observational cohort data.

Our specific research aims were: (1) to estimate the incidence of invasive anal cancer (IAC) and case-survival before (1995-2000) and after (2001-2005) screening program implementation and (2) to examine potential screening program quality indicators. We hypothesized that screening-prompted early surgical intervention for IAC would reduce the incidence of IAC requiring treatment with chemoradiation (IAC_chemorad_).

## METHODS

### Incidence Analysis

The study cohort included all patients under care for HIV infection between 1995-2005 at UCSD Owen Clinic, a multidisciplinary academic adult HIV clinic. Follow up time for each patient began on the date of the first clinic visit during the study period or on 1 January 1995 for those already under care. Follow up time ended on the date of the first

diagnosis of invasive anal cancer (for those developing the outcome) or on the latest of either the date of the last clinic visit during the study period or the end of the study period (for those with visits after 31 December 2005). During the study period, treatment for high grade dysplasia was not routinely offered. All cases of biopsy confirmed invasive anal squamous cell carcinoma were ascertained by review of the clinic electronic medical record, review of surgical pathology records and the medical center cancer registry. Carcinoma in situ (CIS) was classified as a precursor lesion, not as an outcome. Routine anal cytologic screening of all patients under care was implemented as part of a comprehensive anal dysplasia screening program in 2001. Routine human papilloma virus (HPV) typing was not included among the screening procedures. Person-time incidence rates (IR) of IAC were estimated for the pre-screening (1995-2000) and post-screening (2001-2005) periods. Cases of IAC were further classified by primary treatment modality (surgical excision or chemoradiation). Because no treatment for high grade dysplasia was used during the study period, it would not be expected that screening *per se* would reduce the overall incidence of IAC. To estimate the potential impact of screening on IAC_chemorad_, the preventive fractions in the population and in those exposed to screening were estimated for the screening period 2001 – 2005. The preventive fraction for the population (PFp) is defined as the net proportion of all potential cases of IAC_chemorad_ that would be prevented by screening-prompted early surgical intervention. The preventive fraction among the exposed (PFe) is the net proportion of all potential cases of IAC_chemorad_ in the screened population that were prevented by screening [8, 9]. Because of biases inherent in the estimation of preventive fractions from observational studies, we estimated them using two different reference rates of IAC_chemorad_ in the absence of screening: (1) IR _1995 – 2000 _and (2) IR _unscreened, 2001-2005_.

### Case Survival Analysis

Kaplan Meier survival was estimated using two alternative definitions of the origin of time at risk (t0). In one analysis, t0 was taken as the date of diagnosis of IAC. In the alternate analysis, t0 was taken as the date of clinic entry or the opening date of the study period (if visits occurred prior to that date), irrespective of when subsequently the patient was diagnosed with IAC. These analyses were chosen to illustrate the sensitivity of inference regarding case survival to *lead time bias* and *length biased sampling *[10].

### Quality Indicator Analysis

Five potential program quality indicators were examined: (1) screening coverage; (2) percent technically unsatisfactory anal cytology results; (3) cyto-histologic agreement at HRA; (4) time delay between first abnormal anal cytology and first HRA; (5) time between last clinic visit and last anal cytology. *Screening coverage* is defined in this study as the proportion of the target population screened at least once during the screening period [11]. During the study period, cytologic specimens were obtained using the previously described “blind sampling” technique[12] with a moistened Dacron swab and conventional formalin slide fixation. During the study period, the clinic guideline recommended annual anal cytologic examination for all patients[13] and referral to HRA for any cytologic abnormality [7]. Because of limited availability of trained HRA clinicians, patients were triaged to HRA according to severity of the antecedent cytologic abnormality. For example, a high grade (HSIL) or “atypical squamous cells, cannot rule out high grade” (ASC/H) result took priority in scheduling over either low grade squamous intraepithelial lesion (LSIL) or atypical squamous cells of uncertain significance (ASCUS) results. Quality of individual clinician performance of anal cytologic examination was estimated as the percent of technically unsatisfactory results as determined by the reading cytopathologist. Spearman rho was calculated to determine if there was an association between experience (number of cytologic specimens submitted) and the proportion of technically unsatisfactory cytology results. HRA operator accuracy as a measure of procedural quality was estimated by calculating kappa agreement[14] between the most severe histopathologic biopsy diagnosis and the concurrent cytology diagnosis obtained at HRA. For purposes of kappa agreement analysis, cytology results were binary coded as either high grade squamous intraepithelial lesion (HSIL) or lesser abnormality (including low grade SIL, ASCUS and “no atypical or malignant cells”), and histopathologic results were coded as either HSIL (including moderate or severe dysplasia or carcinoma) or lesser abnormality. Following revision of the Bethesda staging system for cervical cytology in 2001[15], an additional cytologic category was created: atypical squamous cells, cannot exclude HSIL (ASC/H). This cytologic abnormality was coded as HSIL for analysis of cyto-histologic agreement. In order to monitor the program so that patients with abnormal results are evaluated in a timely fashion, the two measures of procedure delay were examined overall and stratified according to severity of antecedent cytologic diagnosis. Differences in median procedure delay were evaluated using the Kruskal-Wallis test. The last examined potential quality indicator was program coverage, defined as the proportion of patients under care during the study period that underwent anal cytologic screening at least once.

Statistical analyses were performed using Stata 9.2 (Stata Corporation, College Station, Texas). This study was approved by the UCSD Human Subjects Committee (Project No. 040394).

## RESULTS

### Incidence Analysis

The study cohort included 5,083 patients contributing 13,411 person-years (p-y) at risk between 1 January 1995 and 31 December 2005. Demographic and clinical characteristics of the study cohort have been previously published [5]. The median (IQR) duration of follow up time was 1.8 (0.5 – 4.7) years. During this period, 28 cases of biopsy confirmed IAC were observed, of which 11 were diagnosed in the pre-screening period (1995-2000) and 17 in the screening period (2001-2005). Of the 17 cases diagnosed in the screening period, 10 (59 %) had undergone prior anal cytology screening. Of the 10 IAC patients who had undergone prior anal cytology screening, 2 underwent their first screening less than 6 weeks prior to the diagnosis of IAC. During the screening period, of the 17 IAC cases, the percent with IAC_chemorad _did not vary by screening status (66.7% [6/9] among the unscreened and 75% [6/8] among the screened, exact p=1.0). Table **1** presents the person-time incidence rates (per 100,000 person-years) of IAC overall and IAC_chemorad_ for the pre-screening (1995-2000) and screening (2001 – 2005) periods. Also presented are estimated incidence rates among the screened patient population at risk. The IAC incidence rates in the pre-screening and screening periods were 199 and 216 per 100,000 person-years, respectively with an incidence rate ratio (IRR _screening/pre-screening_) of 1.1 (95% exact CI: 0.48 – 2.56). Of the 28 IAC cases, 22 (78.6%) received chemoradiation. The proportion receiving chemoradiation in the pre-screening period was 90.9 % (10/11) compared with 70.6 % (12/17) in the screening period (exact p=0.355). The incidence rates of IAC_chemorad_ in the pre-screening and screening periods were 181 and 152 per 100,000 person-years, respectively, with a corresponding IRR _screening/pre-screening_ of 0.84 (95% exact CI: 0.33 - 2.17). When incidence was estimated only among those who had undergone prior anal cytology screening between 2001 - 2005, the incidence rates were 126 and 94 for IAC overall and IAC_chemorad_, respectively.

The potential impact of screening without treatment of HSIL lesions on incidence of IAC_chemorad_ was estimated by calculating preventive fractions, comparing incidence among those screened to those not screened using two different reference rates for the unscreened population: (1) IR _1995 – 2000 _and (2) IR _unscreened, 2001-2005_. The IRR_ 2001-2005/1995-2000_ was 0.52 (95% exact CI: 0.16 – 1.58). The corresponding estimated preventive fractions among those exposed to screening (PFe) and in the population (PFp) were 0.48 (95% exact CI: -0.58 - +0.84) and 0.26, respectively. The IRR _screened/not screened, 2001-2005_ was 0.24 (95% exact CI: 0.06 - 0.89). The corresponding estimated PFe and PFp were 0.76 (95% exact CI: 0.11 - 0.94) and 0.62, respectively.

### Case Survival Analysis

Figs. (**1**,**2**) present Kaplan Meier survival estimates for the 28 IAC cases, stratified by screening period and screening status (1995-2000 _pre-screening_, 2001-2005 _unscreened_, 2001-2005 _screened_). In Fig. (**1**), time at risk (t0) was taken as the date of IAC diagnosis. The log rank p-value for equality of the three survival curves was 0.03. In Fig. (**2**), t0 was taken as the date of first clinic visit during the study period (or the opening of the study period if visits occurred prior to that date). The log rank p-value under this assumption was 0.015. Under either assumption of origin of risk time, those in the pre-screening period clearly faired the most poorly, while any suggestive difference between groups during the screen-

ing period was attenuated by assuming t0 to be at clinic entry rather than at IAC diagnosis date.

### Quality Indicator Analysis

**Fig. (3):**
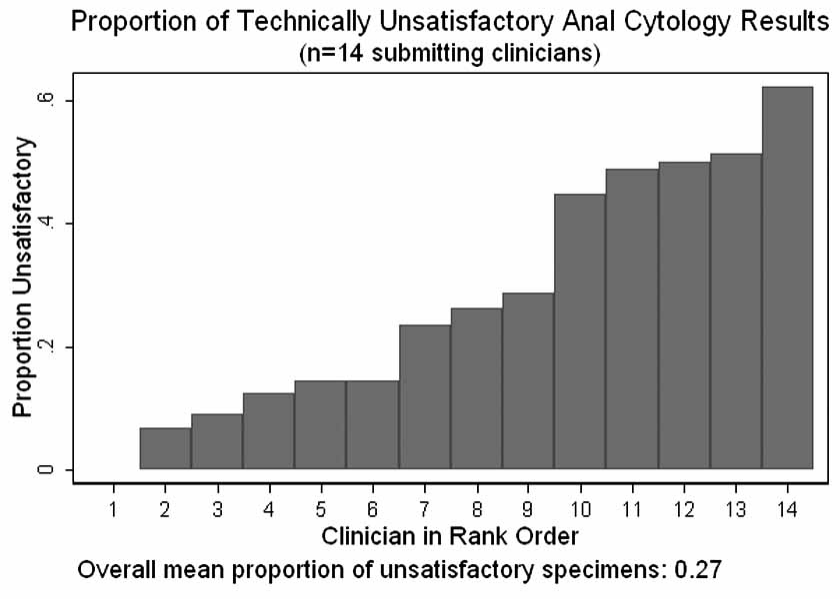
Proportion of technically unsatisfactory anal cytology results.

Overall screening coverage during the screening period was 73%. Fourteen clinicians obtained specimens for anal cytologic analysis during the study period. The median number of specimens submitted per provider was 270, varying from 45 to 839. Among the 14 clinicians, the median percent of specimens read as technically unsatisfactory was 25% but varied from 0 – 62% (Fig. **3**) with no correlation between the number of cytologic specimens submitted by each clinician and the proportion of technically unsatisfactory results (Spearman rho =-0.0022, p=0.99).

**Fig. (4):**
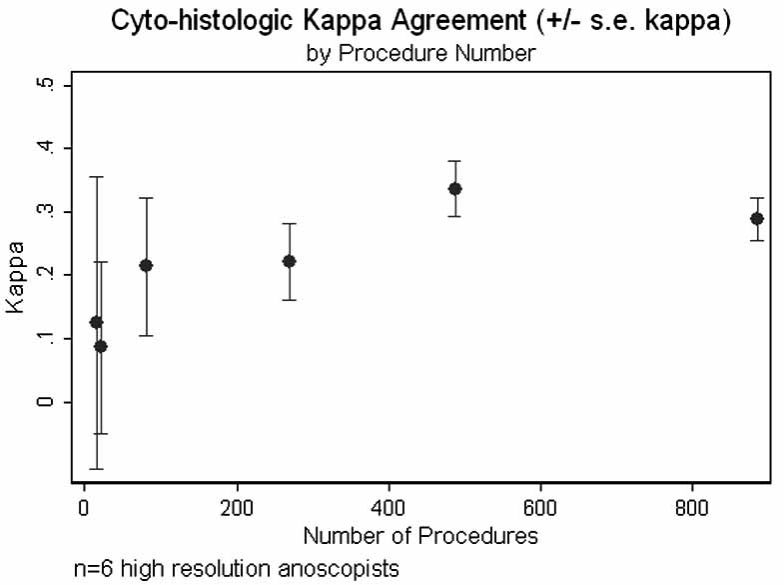
Agreement between anal cytology and anal biopsy histopathology.

Six clinicians performed a total of 1763 high resolution anoscopies between 2001 - 2005. The median number of procedures per operator was 176, varying from 16 – 886. Overall chance-corrected cyto-histologic agreement (kappa) was 0.29, but varied among operators from 0.09 – 0.34. In contrast to what was observed for the technical unsatisfactory cytology indicator, there was a definite relationship (Fig. **4**) between operator experience and kappa cyto-histologic agreement (Spearman rho 0.89, p= 0.02).

The median interval (range) between first anal cytologic examination and first HRA for those with any cytologic abnormality was 258 (1 – 1567) days. This interval varied according to the severity of the first reported anal cytology: 46 days (HSIL or ASC/H), 189 days (LSIL), and 503 days (ASCUS) (Kruskal-Wallis p = 0.0001). The median interval (range) between the last anal cytology and the last clinic visit was 207 (0 – 1639) days. This interval varied by severity of the antecedent anal cytology: 235 days( HSIL or ASC/H), 433 days (LSIL), 1305 days (ASCUS), and 393 days (no atypical or malignant cells) (Kruskal-Wallis p = 0.0001).

## DISCUSSION

### Is Screening Justified?

This description of selected early outcomes and process indicators of a comprehensive screening program for anal squamous cell carcinoma in a population of HIV infected adults under care should be viewed in the context of a generally accepted framework of screening for chronic diseases. Such a framework includes satisfaction of several requirements: (1) The disorder should be well defined with known

prevalence; (2) its consequences should be medically important; (3) an effective remedy should be available; (4) the screening procedures should be simple and safe and should have known and acceptable operating characteristics; (5) the screening program should be cost-effective, (6) implementable in an equitable manner; and (7) the screening procedures should be acceptable to those screened [16]. Of these criteria, there is convincing epidemiological evidence that among HIV-infected men having sex with men, the incidence of IAC is substantial and increasing [17-20], and that its consequences in terms of morbidity and mortality are medically important [3, 21, 22]. The rates of IAC reported in the current study, spanning the first 10 years of potent antiretroviral therapy, are higher than that reported in a cohort of HIV infected patients observed during the period 1996-2003 (92 per 100,000) [23] and comparable to a recent report of IAC incidence among patients with AIDS living in San Diego County between 1996-2000 (144 per 100,000) [19]. There is also evidence that screening procedures thus far recommended [24], based as they are on the model of cervical cancer screening, are relatively simple and safe, with operating characteristics not dissimilar from those reported for cervical cancer screening [5]. Although the addition of HPV typing to cytology is increasingly associated with improved cervical cancer screening program characteristics [25, 26], its role in screening for anal cancer precursors, especially among HIV infected patients, is uncertain. There is some evidence for cost-effectiveness of screening for anal cancer precursors, although the results were sensitive to the assumed rate of progression from precursor lesions to IAC and to the effectiveness of treatment for pre-cancerous lesions [13]. In the case of cervical carcinoma, the most analogous disease process for which screening is universally accepted, the link between precursor lesions and invasive cancer has been established, justifying these precursor lesions as legitimate intermediate targets for detection and intervention [27-31]. However, the relationship between comparable precursor lesions and IAC, while highly likely based on biological similarities, is less well characterized [32-34]. In addition, while several treatment modalities have been suggested for management of anal squamous intraepithelial lesions (ASIL), none have been demonstrated to alter natural history in a conclusive way [3]. We are unaware of any published research regarding the acceptability and psychosocial consequences of procedures employed in anal cancer screening, but there are published models regarding how to address this issue in general and in the context of other disease processes [35-37]. Recent survey data suggest that knowledge of the importance of anal cancer, its association with HPV, and available screening modalities among those at risk may be quite limited [38].

The gold standard for evaluation of screening programs is the randomized controlled trial, but observational designs including both case-control and cohort study designs have contributed to evaluation of screening strategies [39, 40]. The efficacy of cervical cancer screening programs on incidence of and mortality from invasive cervical cancer was based on observational cohort and ecological studies [41, 42]. Because screening for IAC is increasingly practiced at centers treating patients at increased risk for IAC based on existing epidemiological studies, it is worthwhile to consider how such screening programs could be evaluated and what studies should be undertaken to evaluate screening efficacy [10, 43].

### For What Should We Screen?

A distinction should be made between *screening for precursor lesions* to IAC and *screening for early IAC*. In the case of precursor lesions, using estimates from an overview of natural history studies of cervical dysplasia as a model for AIN, the probability of progression of CIN 3 to invasive cervical cancer (ICC) averaged 12 % with, however, a 33% probability of CIN 3 regressing to less severe lesions [31]. A more recent meta-analysis estimated the 6 month transition probability of HSIL (including CIN 2 and CIN 3) to ICC to be 0.0037 (95% prediction interval: 0.00004 –0.03386) [27]. Robust estimates of transition probabilities from HSIL to ICC for HIV infected women are not available although there is evidence that, relative to women without HIV infection, transition probabilities from lower grade to higher grade dysplasia are higher, especially for those with low CD4 count; and regression probabilities from higher to lower grade abnormalities were lower [44]. In designing a screening program that targets identification and treatment of precursor lesions of ICC or IAC, the number needed to screen (NNS) [45, 46] to prevent one targeted outcome (e.g. death, IAC, advanced IAC) will vary as a function of transition probabilities, accuracy of screening procedures, effectiveness of treatment of precursor lesions (including recurrence rates and prognosis after treatment). In contrast to screening for precursor lesions, screening for early IAC without treatment of precursor lesions, although reducing the number of patients undergoing intervention who may never have progressed anyway, runs the risk of intervening too late if screening intervals are too long or screening procedures less than completely accurate.

### Direct and Indirect Measures of Screening Program Success

Potential outcomes for evaluation of a screening program for either precursor lesions or early IAC include, among others[10, 47]: (1) overall mortality rate or mortality attributable to IAC in the population at risk; (2) incidence of all IAC or of advanced IAC; (3) metrics of quality-adjusted survival with IAC; (4) case survival rate; and (5) *stage shift* in presentation with IAC. Of these potential endpoints, Prorok concluded that “there is only one outcome variable known to be valid: the cancer mortality rate”, defined as “the number of cancer deaths per unit of time, per unit of population at risk.”[10] The pretreatment prognosis for IAC is determined, in part, by TNM stage, location, cell differentiation, and comorbid conditions including HIV related immunosuppression [48-51]. Practice guidelines of the National Comprehensive Cancer Network [2] recommend initial local excision for stage T1,N0 (≤ 2 cm diameter, no regional lymph node metastases) anal margin carcinomas and chemoradiation for T1-2, N0 disease for anal canal carcinomas or anal margin carcinomas with positive margins at resection. While it remains controversial whether ablative treatment for precursor ASILs should be routinely offered in the absence of randomized controlled trial evidence of efficacy in reducing the incidence of invasive disease[52, 53], we would argue that an acceptable outcome of a screening program for anal cancer could be detection of disease at a stage no higher than T1N0 if it would permit primary treatment by local resection and spare patients the morbidity associated with chemoradiation. Even if chemoradiation was required, detection of invasive disease at earlier stages should result in more favorable prognosis [54, 55]. Such an early stage endpoint could be viewed as an *indirect measure* or *surrogate marker* of screening efficacy, resulting in a “shift (toward less advanced disease) in the stage distribution of cases detected by screening compared with clinically detected cases.”[16] Validation of stage shift as an indirect outcome measure, however, requires distinguishing prolongation of life due to early treatment from simply extending the *lead time* (the interval between diagnosis at screening and when it would have been detected due to symptoms[47]) with no net gain in survival because treatment had no effect on stage-specific prognosis. Stage shift is additionally vulnerable as an endpoint to what has been termed *overdiagnosis bias *[10] resulting from the identification of early stage invasive disease (e.g. microinvasive disease[56]) that might not have progressed anyway.

Does the early data from our screening program show any evidence of such a shift toward less advanced disease? Only a randomized control trial comparing screening to no screening can definitively address the question because of biases inherent in observational studies such as our own. It would be expected that initially the overall incidence of IAC might increase in the immediate post screening period due to earlier detection of prevalent cases and to progression of sub clinical to clinical disease among those observed in both the pre-screening and screening periods of our study. With regard to stage of disease at presentation, it is likely that screening would tend to detect preferentially early stage disease with longer pre-clinical durations rather than advanced disease that is more likely to present with symptoms. This phenomenon has been termed *length-biased sampling*[16, 57]. Using chemoradiation as a proxy of disease stage, although we observed no significant difference between the proportion of IAC_chemorad_ cases comparing screened and not screened during the implementation phase of the screening program (2001 – 2005), there was a significant difference in the rates of IAC_chemorad_ comparing the screened and not screened during the same period (94 *vs* 395 per 100,000 p-years). This consideration illustrates the extreme caution that must be used in interpreting the statistically significant although likely biased estimate of IRR _screened/not screened, 2001-2005 _of 0.24 reported above. The estimates of prevented fractions based on the same rates are similarly suspect. The estimates of program impact based on the reference rate of IAC_chemorad,1995-2000_ = 181 per 100,000 p-years did not support a contention of shift in disease stage due to screening.

Case survival has been considered a possible endpoint for studies of screening efficacy but its interpretation is subject to both *lead time bias* and *length biased sampling *[10]. Prorok has maintained that distinguishing real increases in case survival attributable to screening from artifactual prolongations in apparent survival due to these biases is “virtually impossible”[10]. However, considering survival time from study entry instead of from IAC diagnosis date would tend to reduce lead time bias by assigning all cases a comparable time at risk origin independent of both screening and diagnosis of IAC. However, if patient entry to the clinic was in any way associated with risk of having subclinical IAC, the comparability of risk at t0 would be compromised. The analyses presented in Figs. (**1**,**2**) illustrate that the decision regarding assignment of t0 in case survival analysis is nontrivial.

### What Should Be the Screening Interval?

Because AIN, like CIN, is a dynamic process with incompletely defined natural history and because the sensitivity of a single cytology and HRA-directed biopsy is too low to preclude an important risk of false negative results, repeat screening at defined intervals is required. In the only published cost-effectiveness study of screening for AIN in HIV infected homosexual and bisexual men, Goldie *et al*. found screening annually or every 2 years to be cost-effective, although the results were sensitive to the rate of progression of ASIL to invasive cancer and to the effectiveness of treatment [13]. It is important to note that one of the assumptions of their model was that there was no shift to earlier stage disease as a result of screening. After a negative baseline screening procedure, the incidence of IAC will increase due to false negative screening tests and development of de novo disease. In the case of cervical cancer screening programs, two baseline cytology examinations are recommended to reduce the false negative rate. The incidence curve after one or more negative baseline screenings will be a measure of the duration of the detectable preclinical phase of disease, the *sojourn time *[58]. Both case-control and cohort studies have been performed to estimate optimal re-screening interval [59]. The definition of a negative screen is itself not straight forward when the screening test result can be viewed as either continuous (e.g. PSA test for prostate cancer) or ordinal (as in cervical and anal cancer screening). Taking into account the imperfect operating characteristics of both cytology and HRA as screening modalities and the cytology trigger used to refer for HRA (e.g. ASCUS or more abnormal), several combinations of results could define negative tests (e.g. 2 negative cytologies or ASCUS cytology with negative HRA). An additional complication, as in our study, is the limited duration of follow up of individual patients in a dynamic cohort. In our study, the median time at risk was 1.8 years, so estimating rates of incident cancer at increasing times after a negative baseline screen becomes less precise as fewer patients are under observation. The results we have presented, however, are based on being screened one or more times during the follow up period and therefore cannot directly address the important issue of optimal re-screening interval. Other programmatic concerns regarding re-screening relate to the follow up of patients already known to have abnormal cytology. In the same clinic population, we showed that the prevalence of AIN 3 at HRA-directed biopsy was 21% and 27% for ASCUS and LSIL cytology results [5]. Evidence-based guidance regarding optimal frequency of interval examination for those with abnormal cytology is lacking.

### Metrics of Screening Program Quality

Separate from consideration of screening program outcome indicators, the ultimate success of screening programs depends on how they are implemented and hence on process indicators of program quality. Such indicators include the achieved *coverage rate* of the target population, maintenance of accuracy and reproducibility of screening procedures, measures of delay and access to both screening and treatment modalities. Although we achieved an overall coverage of 73% for at least one anal cytology screening, close to the 80% benchmark accepted for cervical cytology screening[60], there was a substantial delay in access to high resolution anoscopy especially for those with lower degrees of cytologic abnormality. This delay was attributable both to limited availability of trained HRA operators and to high “no show” rates among scheduled patients. In addition, the interval between last anal cytology and last clinic visit can be interpreted as an additional indicator of program fidelity. The clinic guideline is annual cytology screening for all patients and this target was approximately achieved for all cytology result categories except for ASCUS (median interval 1305 days). Previous research from the study clinic estimated that the prevalence of AIN 3 at biopsy among those with ASCUS cytology was 21%[5], both justifying referral for HRA and indicating the advisability of regular follow up of such patients. The optimal frequency for repeat screening to reduce important IAC-related endpoints has not been determined and likely depends on factors similar to those reported for cervical cancer: the duration of pre-clinical disease, the progression and regression rates of precursor lesions, the sensitivity of and costs associated with screening tests, and the stage-specific curability of detected disease [61, 62]. In the case of HIV-infected patients, there is evidence that progression rates to high grade cervical and anal SIL are higher than among uninfected patients, and that among HIV infected patients, progression rates are higher among the immunosuppressed [63, 64]. In addition, there is evidence that the pathogenesis of the transition from AIN 3 to ICC may differ according to HIV infection status [32]. Therefore optimal SIL stage-specific screening frequency will likely differ according to these risk factors for progression.

With regard to ongoing assessment of clinician technical performance of screening procedures, we examined the percent technically unsatisfactory cytology results as a measure applicable to all primary care providers in the clinic and the agreement between cytology diagnostic category and histopathologic diagnosis as an indicator of high resolution anoscopist technical quality. As Fig. (**3**) demonstrates, there was substantial and clearly unacceptable variability in the proportion of technically unsatisfactory cytology specimens obtained by our fourteen primary care providers. Switching from conventional slide cytology preparation to liquid media offers a technology-based approach to reducing both the variability and rate of unsatisfactory results[65, 66], but ongoing monitoring and training is required to regain an overall technically unsatisfactory rate of 6%[5], that observed during the early period of program implementation at our clinic.

With regard to technical performance of high resolution anoscopists, we evaluated cyto-histologic agreement as a quality indicator and demonstrated a positive relationship between operator experience and kappa agreement. We believe this metric of chance-corrected agreement [14, 67] can be used to compare performance of HRA operators whose patient populations may differ in prevalence of high grade lesions. An alternative indicator, agreement between visual impression and histology, has been evaluated in the context of cervical colposopic accuracy using the Reid index[68], which has not been validated for use in high resolution anoscopy. We are aware of only one publication providing estimates of predictive value of high resolution anoscopic visual findings (e.g. punctation and mosaicism) for high grade dysplasia on biopsy [69]. Standards for proficiency in HRA have not been established. However, based on precedent for training and evaluation of competency in the performance of colposcopy, formal didactic training followed by a clinical mentorship involving supervised performance of 25 – 50 procedures and including at least 10 HSIL cases would be reasonable [70, 71]. Recently the American Society for Colposcopy and Cervical Pathology (ASCCP) has offered courses in performance of HRA (http://www.asccp.org/index.html).

A number of limitations of our analysis, particularly with regard to the potential biases in estimating IAC incidence rates in the two study periods and their associated preventive fractions, as well as the limited duration of follow up, have been discussed above. Additional limitations include: (1) incomplete case ascertainment as a result of loss to follow up; (2) possible selection bias in offering and accepting screening; and (3) possible overdiagnosis bias if some of the early stage IAC cases may not have progressed. The analyses were presented to illustrate approaches to evaluation of evolving screening programs for IAC and its precursor lesions in HIV-infected patient populations.

## CONCLUSIONS

We believe that there is insufficient evidence at the present time to recommend comprehensive screening with cytology followed by referral for HRA and then ablative treatment of high grade lesions as a general practice guideline. It must be recognized that such a comprehensive approach, modeled as it is on the highly successful cervical cancer screening paradigm, could not be widely implemented in the current environment because of very limited numbers of trained HRA operators who would have to split their time between diagnostic and therapeutic procedures. The utility of adjunctive reflex anal HPV testing as a screening component in HIV infected populations, while recommended for cervical ASCUS, is an open research question for which minimal data is available [72]. However, while awaiting further evidence that treatment of precursor lesions favorably alters natural history at acceptable costs, a more limited screening program could be advocated in contrast to doing nothing to detect potentially curable IAC in populations at known high risk. Such a limited program might involve routine cytology screening accompanied by digital rectal examinations and referral either to HRA or a surgeon for any palpable lesions, bleeding, or other anorectal symptoms.

## Figures and Tables

**Fig. (1):**
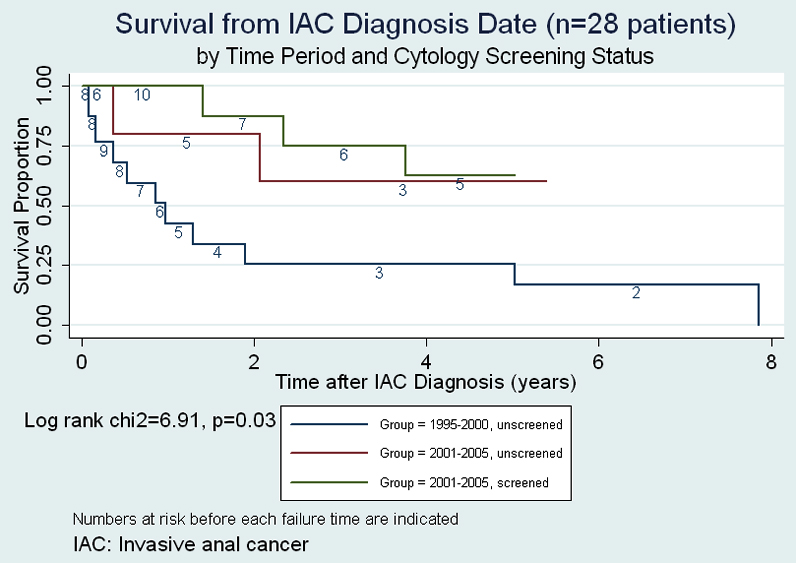
Survival from invasive anal cancer (IAC) diagnosis date.

**Fig. (2):**
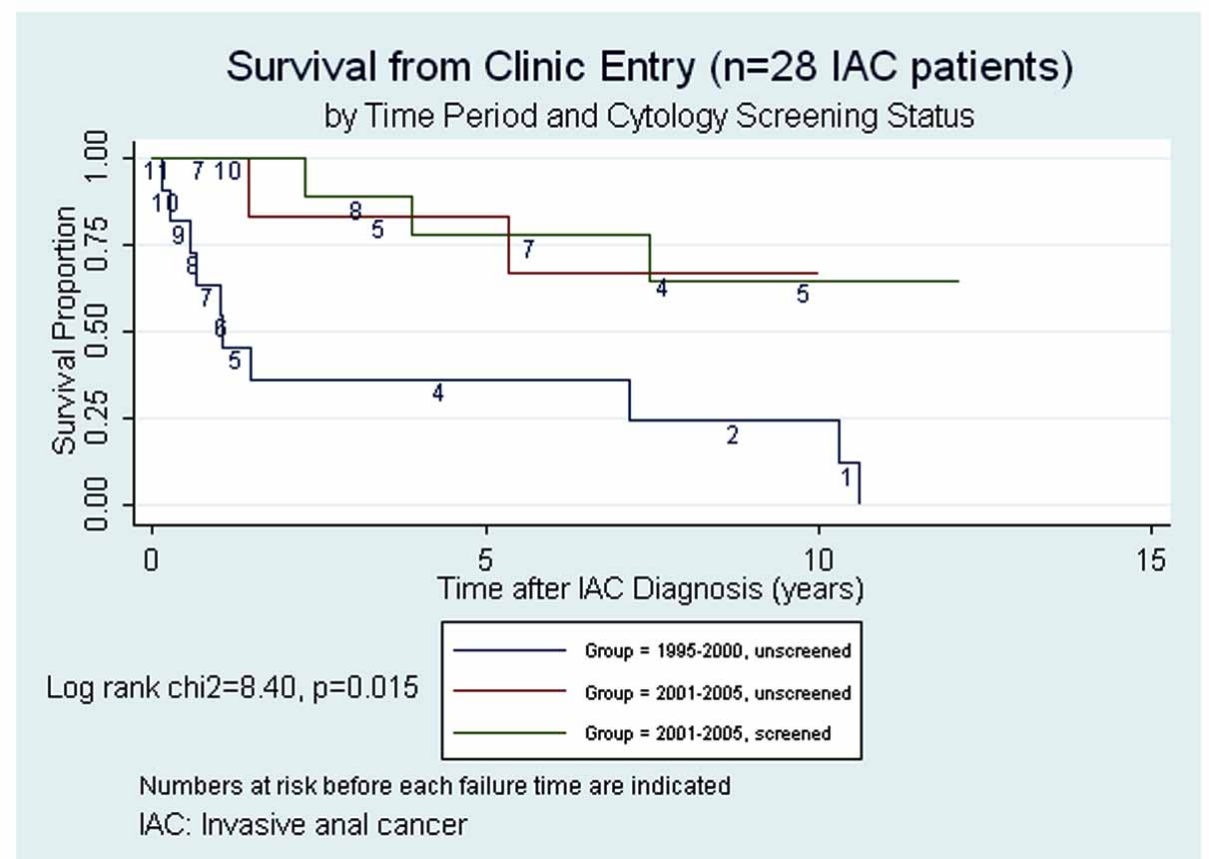
Survival from clinic entry.

**Table 1.** Person-Time Incidence Rates of Invasive Anal Cancer (IAC), by Study Period and by Treatment Modality

	**Pre-Screening Period (1995 – 2000)**		**Screening Period (2001 – 2005)**	
	**Incidence (Per 100,000 Person-Years)**	**95% CI**	**Incidence (Per 100,000 Person-Years)**	**95% CI**
**Any IAC**	199	110 - 359	216	134 - 347
**Any IAC (screened population only)**	N/A	N/A	126	63 - 251
**IAC with chemoradiation**	181	97 - 336	152	86 - 268
**IAC with chemoradiation (screened population)**	N/A	N/A	94	42 - 210
**IAC with chemoradiation (unscreened population)**	N/A	N/A	395	177 - 879
